# Protein arginine methylation/demethylation and cancer

**DOI:** 10.18632/oncotarget.11376

**Published:** 2016-08-18

**Authors:** Coralie Poulard, Laura Corbo, Muriel Le Romancer

**Affiliations:** ^1^ Department of Biochemistry and Molecular Biology, University of Southern California Norris Comprehensive Cancer Center, University of Southern California Los Angeles, Los Angeles, CA, USA; ^2^ Université de Lyon, Lyon, France; ^3^ Université Lyon 1, Lyon, France; ^4^ Inserm U1052, Centre de Recherche en Cancérologie de Lyon, Lyon, France; ^5^ CNRS UMR5286, Centre de Recherche en Cancérologie de Lyon, Lyon, France; ^6^ Equipe Labellisée, La Ligue Contre le Cancer, Paris, France

**Keywords:** PRMT, JMJD6, methylation, demethylation, cancer

## Abstract

Protein arginine methylation is a common post-translational modification involved in numerous cellular processes including transcription, DNA repair, mRNA splicing and signal transduction. Currently, there are nine known members of the protein arginine methyltransferase (PRMT) family, but only one arginine demethylase has been identified, namely the Jumonji domain-containing 6 (JMJD6). Although its demethylase activity was initially challenged, its dual activity as an arginine demethylase and a lysine hydroxylase is now recognized. Interestingly, a growing number of substrates for arginine methylation and demethylation play key roles in tumorigenesis. Though alterations in the sequence of these enzymes have not been identified in cancer, their overexpression is associated with various cancers, suggesting that they could constitute targets for therapeutic strategies. In this review, we present the recent knowledge of the involvement of PRMTs and JMJD6 in tumorigenesis.

## INTRODUCTION

Protein methylation was first described in early 1960s, with the discovery of ε-N-methyl-lysine in the flagellar protein of Salmonella typhimurium [1]. The methylation of lysine residues of histones, was subsequently unveiled several years later [2]. At the beginning of the 1970s, protein methylation of arginine residues was also uncovered by different groups [3–5]. This post-translational modification (PTM) is catalyzed by a family of enzymes called protein arginine methyltransferases (PRMTs). The first enzyme, namely PRMT1, was identified at the end of the 1990s [6], twenty years after the first observation of arginine methylation. During the decade that followed, controversy arose over the reversibility of methylation, since no demethylases had been identified. Indeed, some authors described methylation as a static and stable mark [7–9], opposed to those that believed in a cyclic and transient process [10, 11]. This initial controversy was suspended with the discovery of deiminase enzymes, which catalyze the demethylimination reaction and convert arginine residues into citrulline [12, 13]. However, these enzymes are not regarded as demethylases, since they convert unmodified and monomethylated arginine into citrulline, instead of actually producing a non-modified arginine residue. This controversy finally ended in 2007 when the first and still unique arginine demethylase, namely Jumonji domain-containing 6 (JMJD6), [14] was identified. Nevertheless, the function of this novel enzyme also rapidly became a matter of controversy, and it was finally ascribed two distinct functions, namely as an arginine demethylase [14] and a lysyl-hydroxylase [15].

In the present review, we will summarize the current knowledge on arginine methylation/demethylation, including the controversy on the bifunctional JMJD6. In addition, we will particularly focus on the link between these processes and cancer. Indeed, although arginine methylation is associated with the regulation of many biological pathways (see Table 2, for review [16, 17]), such as signal transduction, protein localization, gene expression regulation, DNA damage repair and RNA metabolism (for review [18, 19]), several studies have demonstrated that PRMTs are involved in carcinogenesis and metastasis (for review [20]). Furthermore, even though JMJD6 was identified only recently, its implication in tumorigenesis has also been reported [21–26].

## ARGININE METHYLATION

### The enzymatic activity of PRMTs

Over 1% of genes in the mammalian genome encode general methyltransferases [27]. Around 0.5% of arginine residues were found to be methylated in mammalian tissues [28]. So far, nine PRMTs have been identified (Table 1). Most of them are ubiquitously expressed, except for PRMT8, which is predominantly observed in the brain [29], and regulate numerous cellular processes, such as proliferation or cell differentiation (Table 2). Mice depleted of PRMTs generally display an abnormal embryonic development (Table 1) [30–35]. Notably, the loss of PRMT1 and PRMT5 leads to embryonic lethality confirming their crucial role in the development of the organism [30, 34].

**Table 1:** PRMTs family members

PRMTs	Domains	Family	Knock-out mice phenotype
PRMT1	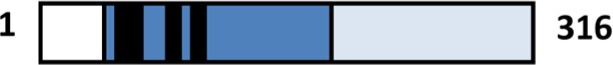	Type I	Embryonic lethal [30]
PRMT2	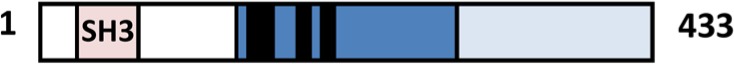	Type I	Viable [31], lean and hypophagic [156]
PRMT3	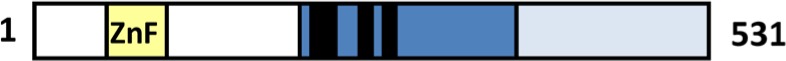	Type I	Embryos small in size but survive after birth [32]
Carm1 (PRMT4)	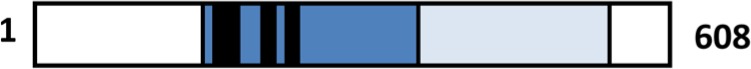	Type I	Newborn mice die shortly after birth [33]
PRMT5	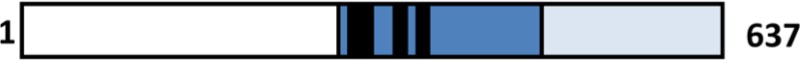	Type II	Early embryonic lethality [34]
PRMT6	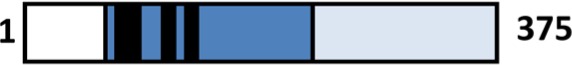	Type I	Viable [35]
PRMT7	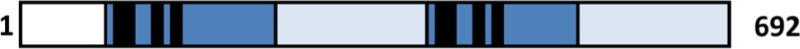	Type III	Reduction in regenerative capacity [157] and abnormality in germinal center formation [158]
PRMT8	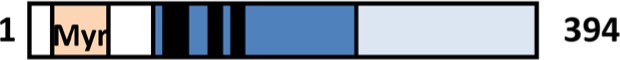	Type I	nd
PRMT9 (FBXO11)	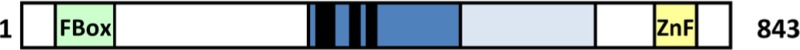	Type II	nd

The catalytic methyltransferase domains (light and dark blue) contain a number of highly conserved motifs (black) that are important for the enzymatic reaction. Additional motifs: SH3 domain (SH3), zinc finger domain (ZnF), myristoylation motif (Myr), FBox motif. nd for non-determined.

**Table 2:** Arginine methylated substrates linked with cancer

PRMTs	Substrates	Effects of arginine methylation	References
**PRMT1**	BRCA1 Mre11, 53BP1 Smad6 Foxo1, Bad ERα H4R3 Axin hnRNPK RUNX1 STAT1 FAM98A INCENP H4R3 EGFR	Affects the tumor suppressor ability of BRCA1. Regulates DNA damage response Initiates BMP-Induced Smad Signaling Impeeds Akt phosphorylation Prerequisite for Akt activation Allows the binding of TDRD3 Stabilization from proteosomal degradation Negatively regulates cell apoptosis Prevent the corepressor SIN3A binding IFNalpha/beta-induced transcription Required for tumor cell migration, invasion and colony formation Promotes mitosis Transcriptional activation of ZEB1 (promotes EMT) Enhances EGF binding, dimerization and signaling activation	[58] [59, 60] [67] [64, 65] [61–63] [68] [66] [69] [70] [71] [72] [73] [56] [74]
**PRMT2**	STAT3	Regulation of leptin signaling	[156]
**PRMT3**	rpS2	Inhibits ubiquitination of ribosomal protein S2 (rpS2)	[159]
**Carm1 (PRMT4)**	H3R17 SRC3, p300/CBP BAF155 SOX2 PAX7 HSP70 Carm1 itself RNA polymerase II MED12	Transcriptional activation of E2F1 (promotes cell growth) Coactivator regulation Enhances tumor progression and metastasis Enhances self-association of SOX2 Allows MLL1/2 recruitment during stem cell division Regulates retinoid acid-mediated RARβ2 gene activation Allows coupling of transcription and mRNA splicing Facilitates the expression of select RNAs Sensitizes human breast cancers to chemotherapy drugs	[90] [83, 84] [92] [160] [161] [145] [162] [163] [93]
**PRMT5**	H3R8, H4R3 E2F1 p53 PDCD4 EGFR CRAF FEN1 HOXA9 RAD9 p65 subunit (RelA) RPS10 SREBP1	Repress expression of tumor suppressor genes (ST7, NM23) Controls growth regulation Control mechanism of p53 response Accelerates tumor growth Contributes to attenuate EGFR-mediated ERK activation Limits the ERK1/2 signal Facilitates PCNA binding Essential for E-selectin induction Required for cellular resistance to DNA damaging stresses Activates NF-κB gene expression Regulates ribosome biogenesis Increases growth of hepatocellular carcinoma	[104, 105] [112] [108] [102] [111] [113] [114] [115] [116] [117] [118] [119]
**PRMT6**	p21CDKN1A HMGA1 DNA polymerase β p16	Cytoplasmic localization / resistance to cytotoxic agents nd Regulates DNA base excision repair Impedes the p16/CDK4 interaction and induces cell proliferation	[122] [164] [165] [123]
**PRMT7**	H2AR3, H4R3	Regulates cellular response to DNA damage	[166]
**PRMT8**	EWS	nd	[167]
**PRMT9**	SAP145	Creates a tudor domain for SMN protein	[168]

BRCA1: Breast cancer gene 1, 53BP1: p53 binding protein 1, BMP: Bone morphogenetic proteins, Foxo1: forkhead box protein O1, Bad: BCL-2 antagonist of cell death, ERα: estrogen receptor alpha, TDRD3: Tudor Domain Containing 3, hnRNPK: Heterogeneous Nuclear Ribonucleoprotein K, RUNX1: Runt-related transcription factor 1, STAT: Signal Transducer And Activator Of Transcription, IFN: Interferon, FAM98A: Family with sequence similarity 98, member A, INCENP: Inner centromere protein, ZEB1: zinc finger E-box binding homeobox 1, EMT: Epithelial-Mesenchymal Transitions, EGFR: Epidermal Growth Factor Receptor, SRC3: steroid receptor coactivator-3, CBP: CREB binding protein, BAF155: BRG1-associated factor 155, SOX: SRY-box, PAX7: Paired box 7, MLL: Mixed Lineage Leukemia, HSP70: heat shock protein 70, RAR: Retinoic acid receptor, MED12: Mediator Complex Subunit 12, PDCD4: Programmed cell death-4, ERK: Extracellular signal-regulated kinases, CRAF: Proto-oncogene C-RAF, FEN1: Flap Structure-Specific Endonuclease 1, PCNA: proliferating cell nuclear antigen, HOXA9: Homeobox A9, RPS10: Ribosomal Protein S10, SREBP1: Sterol regulatory element-binding protein 1, p21 CDKN1A: Cyclin-Dependent Kinase Inhibitor 1A, HMGA1: High Mobility Group AT-Hook 1, EWS: Ewing sarcoma, SAP145: Spliceosome-associated protein 49, SMN: Survival of Motor Neuron.

**Figure 1:**
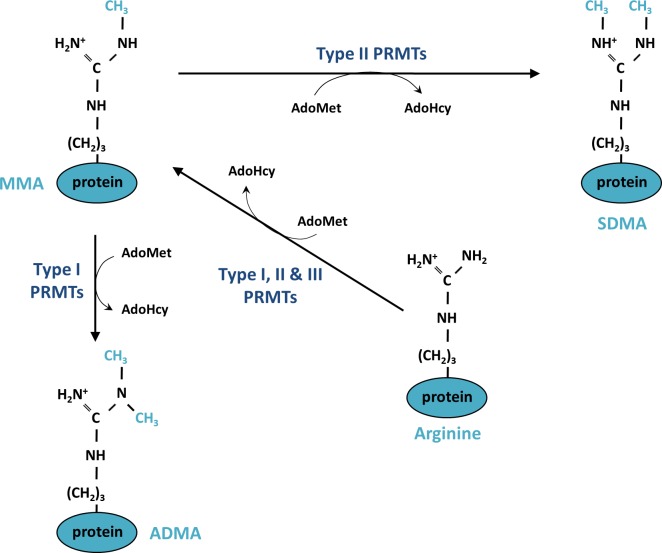
Mechanism of protein methylation on arginine residues Monomethylation (MMA) is catalyzed on one of the terminal guanidino-nitrogen atom of an arginine by Type I, II and III protein arginine methyltransferase (PRMTs). The enzymatic reaction consumes a methyl donor, S-adenosylmethyionine (AdoMet) and generates S-adenosylhomocysteine (AdoHcy). Type I PRMTs generate asymmetric dimethylation (ADMA) and Types II PRMTs are responsible for symmetric dimethylation (SDMA)

PRMTs catalyze the transfer of a methyl group from a methyl donor, S-adenosylmethionine (AdoMet) to a guanidino-nitrogen atom. This reaction generates S-adenosylhomocysteine (AdoHcy) and methylated arginine. Three types of methylated arginine residues are found in mammalian cells; namely, ω-N^G^-monomethylarginine (MMA), ω-N^G^- N^G^- asymmetric dimethylarginine (ADMA) and ω-N^G^- N’^G^-symmetric dimethylarginine (SDMA) (Figure 1). PRMTs are classified into three groups of enzymes (Types I, II and III) according to the type of methylation they catalyze. All of these types produce MMA; additionally, Type I PRMTs (PRMT1, PRMT2, PRMT3, Carm1 (PRMT4), PRMT6 and PRMT8) are responsible for producing ADMA, in which the methyl groups are linked to the same guanidino nitrogen atom, while Type II PRMTs (PRMT5 and PRMT9) lead to the symmetrical addition of the methyl groups on each of the guanidino nitrogen atom of the arginine, resulting in an SDMA (Figure 1). PRMT7 is the only Type III enzyme, exclusively catalyzing the formation of MMA [36, 37].

Both nuclear and cytoplasmic proteins harboring glycine- and arginine- rich (GAR) motifs are the main targets of arginine methylation [38]. Indeed, PRMT1, PRMT3 and PRMT6 mainly recognize and methylate RGG repetitions in GAR motifs; whereas Carm1 preferentially modify arginine residues present in proline-, glycine-, and methionine (PGM) rich regions largely found in splicing factors. PRMT5 modifies substrates containing both GAR and PGM motifs [39], while PRMT7 preferentially methylates substrates with an RXR motif, consisting in a pair of arginine residues separated by one basic residue [37]. As a result of arginine methylation, GAR and PGM methylated motifs interact mainly with proteins *via* their Tudor domains [40]. The human genome encodes over thirty members of these proteins, including SMN, the splicing factor 30 (SPF30) and the Tudor domains of the Tudor domain-containing proteins (TDRD), which are involved in many cellular processes (for review [41]).

### PRMTs and cancer

The aberrant expression of PRMTs and the dysregulation of their enzymatic activity have been associated with several diseases (for review [16, 42]), including many types of cancers. However, exhaustive functional studies are only available for some of them, and although they have been reported in different types of cancers (Table 3), our review will focus on their link with breast cancer.

#### PRMT1

PRMT1 is the major PRMT, modifying around 90% of methylated arginine residues in mammalian cells [43], thereby catalyzing the majority of the ADMA. Upregulation of the protein is a feature of many types of human cancers [44–47], and high levels of PRMT1 mRNA have notably been reported in high grade breast cancers [48–53] (Table 3). Indeed, Goulet et al. found that the level of PRMT1 expression was higher in human breast tumor samples compared to adjacent normal breast tissue [47]. Furthermore, these authors demonstrated that the alternative splicing of PRMT1 pre-mRNA results in seven isoforms of the protein (PRMT1-v1 to v7), which differ in their N-terminal sequence, and are found to be dysregulated in breast cancer. This finding was later corroborated by the same team, which showed that the relative balance of PRMT1 isoforms is altered in breast cancer. For instance, PRMT1-v2 is overexpressed in breast cancer cell lines and mammary tumors and its cytoplasmic localization is linked with its key role in cell survival and invasion of breast cancer cells [54].

We can hypothesize that an overexpression of PRMT1 could trigger a hypermethylation of its substrates, accelerating the process of tumorigenesis. For example, at the transcriptional level, PRMT1 catalyzes the methylation of the R3 residue of histone H4 (H4R3), an epigenetic active mark [55]. H4R3 methylation at the ZEB1 promoter activates its transcription [56]. ZEB1 is a key transcription factor inducing the epithelial-mesenchymal transition (EMT) by repressing the transcription of E-cadherin [57]. Gao et al. demonstrated that PRMT1 is a key regulator of the EMT in breast cancer cells, promoting migration and invasion. The expression of ZEB1 is essential for PRMT1-induced EMT, migration and invasion but also for the acquisition of stem-cell-like properties [56]. Overexpression of PRMT1 may trigger cancer cells to form metastases.

A large number of non-histone substrates for PRMT1 have also been identified. BRCA1 was found to be methylated by PRMT1 in breast cancer cell lines, as well as in breast tumor samples [58]. This methylation event alters the recruitment of BRCA1 to specific promoters. Because of the involvement of its tumor suppressor activity in cell cycle transcription, DNA damage response and chromatin remodeling, a dysregulation in BRCA1 following aberrant methylation could result in genomic instability. Moreover, PRMT1 methylates the DNA repair pathway proteins MRE11 and 53BP1 (p53 binding protein 1). Methylation of MRE11 regulates its exonuclease activity on double-stranded DNA, and is required for DNA damage checkpoint control [59]. Likewise 53BP1, which is involved in DNA damage repair pathways, is methylated by PRMT1. Its methylation was shown to be essential, by following the effects of mutations of key arginine residues targeted by PRMT1 for methylation, which abrogated its binding to single or double-stranded DNA, preventing the recruitment of the DNA repair machinery [60]. Similarly, the inhibition of 53BP1 methylation, achieved by treating cells with methyltransferase inhibitors, disrupts 53BP1 localization to damaged DNA and forms fewer foci [60]. In conclusion, a dysregulation in the activity of PRMT1 could lead to an accumulation of DNA damage, a well-established cause of tumorigenesis.

PRMT1 is also largely involved in cell signaling. For instance, in estrogen signaling, the methylation of the R260 residue of ERα is a key step in promoting the formation of a complex containing methylated ERα/Src/PI3K, and the subsequent activation of the Akt pathway [61, 62]. Of note, this pathway is activated in aggressive breast tumors, and the high level of expression of this complex is an independent marker of poor prognosis, associated with reduced disease-free survival, supporting the idea that ERα hypermethylation could trigger abnormal cell proliferation [63]. Besides, PRMT1 has also been shown to regulate Akt-mediated signaling by other mechanisms, inducing apoptosis. One such example is through the methylation of the Forkhead box O1 (FOXO1), on the R248 and R252 residues in the Akt consensus phosphorylation site, which blocks its phosphorylation on the adjacent S253 residue [64]. This results in the retention of the FOXO1 in the nucleus, preventing its proteasomal degradation, and consequently increasing its transcriptional activity on its targeted genes. Likewise, PRMT1 binds and methylates the BCL-2 antagonist of cell death (BAD) on residues R94 and R96, inhibiting Akt phosphorylation on the S99 residue, which in turn prevents the binding of 14-3-3 protein [65], resulting in apoptosis. Inhibition of PRMT1 thus inhibits apoptosis, which is directly linked with tumorigenesis by promoting cell survival. PRMT1 also regulates the Wnt signaling pathway by methylating the R378 residue of axin, a negative regulator of this Wnt pathway [66]. Methylation of axin enhances its interaction with the glycogen synthase kinase 3β, leading to a decrease in its degradation. As an aberrant regulation of the Wnt pathway is a driver of many different cancers, it is tempting to speculate that a dysregulation in the expression of PRMT1 could be linked with tumorigenesis.

In addition, PRMT1 catalyzes the methylation of many other proteins involved in important cellular processes [67–74] (Table 2); however, further studies are required to determine whether their methylation is linked with tumorigenesis.

#### PRMT2

PRMT2 was discovered through its sequence homology with PRMT1 [75], although no methyltransferase activity was associated with this enzyme at the time. Indeed, PRMT2 was only recently ascribed a Type I arginine methyltransferase activity [76], which appears to be much lower than that of PRMT1, explaining why only a limited number of substrates have been identified so far (Table 2). However, PRMT2 has been shown to enhance the transactivation of nuclear receptors, like ERα, progesterone receptors and androgen receptors, largely involved in the development of hormone-dependent cancers [77, 78]. In effect, the expression of PRMT2 and its splice variants is increased in breast tumors compared to normal tissues. In addition, mRNA expression of PRMT2 is correlated with an ERα-positive status [79]. Zhong et al. confirmed these results at the protein level, but found that the level of nuclear PRMT2 was lower in breast tumors in comparison with healthy tissues [80]. Furthermore, nuclear loss of PRMT2 has been shown to be associated with an increase in cyclin D1 expression and with tumor grade in invasive ductal carcinoma [80].

However, given the very few identified substrates, it is currently difficult to link the enzymatic activity of PRMT2 with tumorigenesis.

#### Carm1 / PRMT4

Carm1 is a Type I enzyme, which catalyzes the ADMA modification. The protein was first identified in a yeast two-hybrid screen, associated with the coactivator GRIP-1, and involved in transcriptional regulation [81]. Carm1 modifies histone H3 on the R17 residue (H3R17me2), as well as coactivators, such as p300/CBP and SRC-3 [82–84]. Several studies described an aberrant expression of Carm1 in hormone-dependent tumors, such as prostate and breast cancers [85, 86], but also in other types of cancers [46, 87–89] (Table 3).

Carm1 was shown to be essential for estrogen-induced cell cycle progression in breast cancer cell lines [90]. Indeed, after estrogen stimulation, nucleosomes associated with the gene encoding the critical cell cycle transcriptional regulator E2F1 are methylated by Carm1 on the R17 residue of histone H3 (H3R17me2), concomitantly with the recruitment of ERα and increased E2F1 gene expression. Furthermore, the recruitment of Carm1 is dependent on the SRC-3 coactivator, which is overexpressed in aggressive breast tumors concomitantly with a Carm1 overexpression [85]. Since the methylation of SRC-3 increases its activity and stability [84, 91], a high level of Carm1 could enhance the expression of genes targeted by SRC-3 and induce cell proliferation in these tumors.

BAF155, a core subunit of the chromatin remodeling complex SWI/SNF, is another Carm1 substrate. Methylated BAF155 is directed on the chromatin of several target genes involved in the c-Myc pathway (*CDCA7, COL1A2, GADD45A, DDX18, and NDRG1*) and is correlated with an increase in their expression. Using an antibody specific for BAF155 methylation, the analysis of a large cohort of breast tumors revealed that BAF155 methylation was associated with cancer progression, malignancy and poor survival. Moreover, BAF155 knock-down in the aggressive breast cancer cell line MDA-MB-231, impaired proliferation and migration, while the methylation of BAF155 promoted *in vivo* metastasis [92].

In addition, a recent study identified the RNA polymerase II mediator complex subunit (MED12), as a new substrate for Carm1, [93]. The methylation of MED12 rendered breast cancer cells sensitive to chemotherapy. These observations were validated in mouse models and in breast cancer patients. Indeed, in a clinical cohort containing 154 patients treated with 5-fluorouracil and doxorubicin, both MED12 and Carm1 protein levels predicted response to chemotherapy. Using RNA microarray analyses, the authors identified the *p21/WAF1* gene among the main upregulated genes, following the depletion of MED12 or Carm1. The methylation of MED12 enhances its recruitment to the *p21* gene locus, in order to suppress the transcription of *p21*, previously described to inhibit drug response [94]. The authors thus identified an unexpected new mechanism involving Carm1-methylation and drug resistance. This study demonstrated that methylation could be a sensor for drug responses in human cancers.

#### PRMT5

PRMT5 is the major Type II methyltransferase that catalyzes SMDA [39, 95, 96]. As was mentioned above for the others PRMTs, upregulation of the protein is associated with various cancers [97–102] (Table 3).

PRMT5 was initially shown to be involved in transcriptional repression events in the nucleus, and in snRNP biogenesis [103] in the methylosome, a large (20S) protein arginine methyltransferase complex located in the cytoplasm [96]. For example, symmetric dimethylation of histone H4 on the R3 residue (H4R3me2s) and on the R8 residue of histone H3 (H3R8me2s by PRMT5) represses the tumor suppressor gene *ST7* (suppression of tumorigenicity 7) [104, 105] and cyclin E1 [106], but also represses the cell cycle regulator CDKN2A (cyclin-dependant kinase inhibitor 2A) [107]. PRMT5 also induces transcriptional silencing by methylating p53 or MBD2 (methyl-CpG binding domain protein 2), altering their biochemical functions [108, 109]. The R110 residue of the tumor suppressor protein PDCD4 (programmed cell death protein 4) is another substrate for PRMT5. In an orthotopic breast cancer model, co-expression of PDCD4 and PRMT5 accelerates tumor growth. The R110 of PDCD4 and the catalytic activity of PRMT5 are necessary for tumor growth. Furthermore, high levels of PDCD4 and PRMT5 expression in breast cancer were correlated with poor patient outcome [102]. Interestingly, methylation of PDCD4 by PRMT5 occurs near a S6 kinase 1 site (S67) reported to regulate PDCD4 stability, and could explain the impact of PDCD4 methylation in tumor growth [110].

Of note, the subcellular localization of PRMT5 changes in transformed cells; it is mostly cytoplasmic in primary and immortalized cells, and it becomes nuclear in transformed cells [104, 105]. The localization of PRMT5 could be involved in controlling cell growth and proliferation. Indeed, PRMT5 methylates EGFR on the R1175 residue, enhancing phosphorylation of the Y1173 residue, which attenuates EGFR-mediated ERK activation [111]. Methylation of R1175 creates a binding site for the phosphatase SHP1, which impedes the downstream EGFR signaling pathway. It is tempting to speculate that a relocalization of PRMT5 in the nucleus decreases R1175 methylation, triggering an increase in EGFR-mediated ERK signaling, inducing proliferation, migration and invasion in the mammary gland.

Additionally PRMT5 methylates numerous other proteins regulating normal cell processes [112–119]. We could speculate that a fine regulation of PRMT5 methylation is important to maintain the proper cell processes.

#### PRMT6

PRMT6 has been reported to be overexpressed in bladder, lung and breast cancer cells (Table 3) [44], and the elevated levels observed in breast tumor samples by immunohistochemistry (IHC) were correlated with tumor stage [120]. However, it was also reported that PRMT6 mRNA levels were lower in breast cancer compared to normal breast tissues [121], a discrepancy that could be due to the small number of samples analyzed in the latter study, as well as the different level of expression examined, mRNA *versus* protein.

PRMT6 substrates potentially linked with cancer have only recently been identified. Indeed, PRMT6 is able to methylate tumor suppressor genes, such as *p21* [122] and *p16* [123]. Methylation of p21 on residue R156 promotes the phosphorylation of T145, resulting in the increase of cytoplasmic localization of p21, inhibiting its growth suppressive function, and making colon cancer cells more resistant to cytotoxic agents [122]. In addition, methylation of p16 disrupts the interaction of PRMT6 with CDK4, thus impeding the blockade of the cell cycle [123].

**Table 3:** PRMTs dysregulation in human cancer

PRMTs	Dysregulation in human cancers	References
**PRMT1-v1**	Overexpressed in breast cancer, bladder cancer, pediatric acute lymphoblastic leukemia and in non-small cell lung carcinomas Upregulated in lung cancer and glioma tissue Associated with poor prognosis of colon cancer	[44–47] [50–53]
**PRMT1-v2**	Overexpressed in breast cancer Upregulated in colon cancer and associated with poor prognosis	[54] [52, 53]
**PRMT2**	Overexpressed in breast cancer and associated with ERα-positive tumors. Overexpressed in breast cancer but decrease localization in the nucleus Low expression in breast cancer compared with normal breast.	[79] [80] [169]
**PRMT3**	nd	
**Carm1 (PRMT4)**	Overexpressed in prostate cancer, colorectal cancer, non-small cell lung carcinomas Early expression in early stages of hepatocarcinogenesis. Associated with poor prognosis in breast cancer	[46, 86, 87] [88] [85, 89]
**PRMT5**	Upregulated in lung, gastric, bladder, colon cancer and lymphoma. Overexpressed in breast cancer and epithelial ovarian cancer Cytoplasmic expression is associated with high-grade subtypes of primary lung adenocarcinomas	[97, 98] [99, 100, 102] [101]
**PRMT6**	Overexpressed in bladder and lung cancer cells Overexpressed in breast cell lines and cancer. Correlation between its expression and tumor stage Downregulated in invasive breast ductal carcinoma	[44] [120] [121]
**PRMT7**	Overexpressed in breast carcinoma cells. Overexpressed in primary breast cancer and breast cancer lymph node metastasis	[125] [126]
**PRMT8**	nd	
**PRMT9 (FBXO11)**	nd	

ERα : Estrogen Receptor alpha, nd for non-determined.

#### PRMT7

PRMT7 was initially characterized as a Type II methyltransferase, but was recently ascribed as a Type III, generating monomethylation as a final step [36, 37]. Although limited information is available on the biological functions of PRMT7, a growing body of evidence has shown that it could be linked with cancer. Indeed, *PRMT7* is localized in a region known to have an aberrant copy number in metastatic breast cancers [124]. Moreover, two independent groups demonstrated that PRMT7 is overexpressed in breast carcinomas (Table 3) [125, 126]. Baldwin et al. showed that PRMT7 induces the expression of matrix metalloproteinase 9, a well-known mediator of breast cancer metastasis [126]. In parallel, Yao et al. reported that elevated PRMT7 mediates EMT through inhibition of E-cadherin expression by epigenetic modification. [125].

In summary, the overexpression of PRMTs participates in promoting oncogenic transformation by triggering the aberrant methylation of substrates involved in tumorigenesis (Table 2).

## ARGININE DEMETHYLATION

The first arginine demethylase activity was unveiled by the group of Bruick in 2007 [14]. The enzyme responsible for this demethylation was JMJD6 (Jumonji domain-containing 6), which had previously been described as putative phosphatidylserine receptor (PSR) [127]. Controversy over its true function thus arose and persisted until recently (for review [128]), and will be discussed in the first part of our review on arginine demethylation, while the second part will focus on presenting new evidence linking JMJD6 and tumorigenesis (Table 4).

### The bifunctional JMJD6

#### From a phosphatidylserine receptor to an arginine demethylase

**Figure 2:**
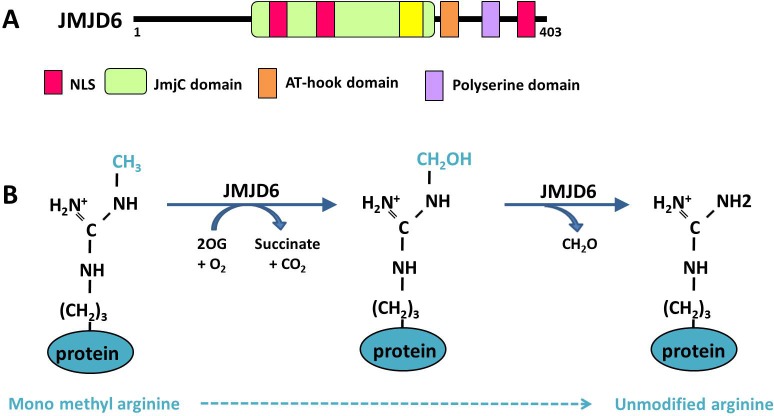
Functional domains and demethylase activity of JMJD6 **A.** The different functional domains of the JMJD6 protein are presented. NLS: Nuclear localization signal. **B.** Demethylation reaction of a monomethylated arginine is catalyzed by JMJD6 within 2 steps. First JMJD6 hydroxylates the methyl group consuming oxoglutarate (2OG), then a deformylation reaction produces formaldehyde (CH_2_O) leading to an unmodified arginine

JMJD6 was originally described as a phosphatidylserine receptor (PSR), at the level of the plasma membrane of macrophages and dendritic cells [127]. Since its discovery in the year 2000, contradictory studies have been published on the function of JMJD6. The most unexpected findings were those revealed by JMJD6 loss-of-function experiments, which were unable to confirm its involvement in the phagocytic process in mice, but showed that transgenic mice carrying JMJD6 null mutations died around birth [129–131]. Two subsequent studies reported a nuclear localization of JMJD6 [132, 133], suggesting that it could be involved in other processes. A bioinformatics analysis of JMJD6 protein in *Hydra vulgaris* led to the identification of three nuclear localization signals (NLS), a JmjC domain and a DNA binding domain (AT-hook domain) (Figure 2A). The Jumonji C (JMJC) domain can catalyze protein demethylation through an oxidative mechanism requiring iron Fe (II) and 2-oxoglutarate (2OG) as cofactors. Moreover, this *in silico* analysis also uncovered a high level of homology between JMJD6 and the FIH-1 protein (factor inhibiting HIF), which possesses a 2OG-and Fe (II)-dependent oxygenase activity [133], suggesting that JMJD6 could display a similar oxidative enzymatic activity (Figure 2B).

Since then, several reports have shown that JMJD6 was mainly localized in the nucleus, with a diffuse expression in the nucleoplasm, as well as in nuclear speckles [15]. Interestingly, these latter structures concentrate numerous methylated proteins [134], in addition to being enriched in RNA splicing factors. Moreover, a pool of JMJD6 is also localized in the cytoplasm and a recent publication indicated that JMJD6 could either be found in the cytoplasm or in the nucleus according to the cellular context [135]. Indeed, it appears that, JMJD6 is localized at the cell surface in immature macrophages, in order to regulate phagocytosis, and then translocates into the nucleus of differentiated cells. Such a mechanism was also unveiled for HER2 that translocate into the nucleus *via* its NLS [136].

#### The enzymatic activities of JMJD6

**Figure 3:**
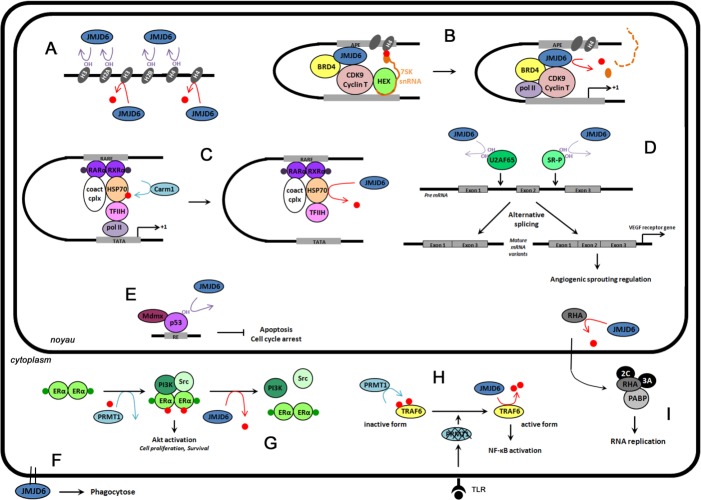
The functions of JMJD6 in cells Red dots represent methylation events, and OH hydroxylation reactions. **A. Epigenetic regulation.** JMJD6 catalyzes the demethylation (red arrow) of histone H3 (H3R2me2) and histone H4 (H4R3me2), and lysine hydroxylation (purple arrow) of histones H3, H4, H2A and H2B. **B. Regulation of transcriptional pause release** on distal anti-pause enhancers (APE) of a subset of transcriptional units. JMJD6 catalyzes H4R3me2 demethylation and RNA demethylation in the cap structure of 7SK snRNA (orange dot), and is responsible for pause release and transcription elongation. **C. Retinoic acid-mediated RARβ2 gene regulation**. Upon RA stimulation (purple dots), RARα, RXRα, HSP70, Carm1 and coactivator complexes (Coact cplx) bind on RARβ2 gene promoter (RARE). Carm1-methylation of HSP70 allows the recruitment of the preinitiation complex (TFIIH) and expression of RARβ2 genes. JMJD6 demethylation reverses this process. **D. Pre-mRNA splicing regulation**. Lysine hydroxylation by JMJD6 of U2AF65 (K15, K276) and SR-like proteins (SR-P) regulates alternative splicing. For example, VEGR receptor gene splicing is regulated by U2AF65 hydroxylation. **E. Repression of p53 transcriptional activity.** JMJD6 catalyzes p53 hydroxylation. This event antagonizes p53 acetylation and promotes the interaction of p53 with the transcriptional repressor Mdmx resulting in inhibition of apoptosis and cell cycle arrest. **F. Phagocytosis regulation**. JMJD6 is localized at the cell surface of immature macrophages in order to regulate phagocytosis. **G. Regulation of estrogen non-genomic action.** Upon E2 stimulation (green dots), ERα is methylated by PRMT1. This event is a prerequisite to the recruitment of PI3K and Src, and the activation of Akt. JMJD6 demethylates ERα, promoting the dissociation of the complex. **H. Control of innate immune responses.** Toll like receptor (TLR) ligands induce PRMT1 degradation. JMJD6 demethylates TRAF6 leading to full NF-κB activation. **I. Regulation of foot-and-mouth disease virus (FMDV) infection**. FMDV infection relocalizes JMJD6 in the nucleus where it will demethylate RNA helicase A (RHA). RHA methylated exits from the nucleus and interacts in the cytoplasm with the replication machinery

As mentioned above, arginine demethylation was first described in 2007, on histone proteins, modifying the R2 and R3 residues of histone H3 and H4, respectively (Figure 3A) [14]. Similarly to the lysine demethylases belonging to the JmjC family, JMJD6 is a JmjC-containing iron- and 2-oxoglutarate-dependent dioxygenase. During a JMJD6-demethylation event, hydroxylation of the methyl group is immediately followed by a deformylation producing formaldehyde (Figure 2B).

Subsequently, some reports challenged the original demethylase activity of JMJD6. Indeed, they demonstrated that JMJD6 also acts as a hydroxylase by catalyzing the lysyl-5-hydroxylation of the splicing regulatory factor U2AF65 (U2 small nuclear ribonucleoprotein auxiliary factor 65-kilodalton), of multiple lysyl residues on histone H3 and H4 tails and of the tumor suppressor p53 (Figure 3A, 3D, 3E) [15, 137–139]. In 2009, Webby et al. demonstrated that the hydroxylation of U2AF65 on the lysine K15 and K276 residues regulates RNA splicing of some specific genes [15]. Different groups then reported the role of JMJD6 in the regulation of RNA splicing [138, 140, 141]. JMJD6 also catalyzes the hydroxylation of lysine residues of histones H2A and H2B [137, 139]. Interestingly these authors showed that hydroxylation of lysine residue inhibits methylation and, inversely monomethylation of lysine residues blocks hydroxylation by JMJD6, suggesting that JMJD6 is essential to maintain the “histone code” [139].

However, even if the demethylase activity of JMJD6 was questioned for almost a decade, the group led by Rosenfeld and others clearly validated this enzymatic activity in 2013 [142–146]. Indeed, this team demonstrated the removal of ADMA and SDMA from H4R3me2 by JMJD6, and described a novel removal of the methyl group from the cap structure of 7SK snRNA on anti-pause enhancers [142] (Figure 3B). In their study, JMJD6 specifically catalyzed the demethylation of H4R3me2 in a panel of modified histones, while the enzymatically inactive JMJD6 mutant (H187A) had no effect on this modification. This result, initially controversial, is now well recognized by the scientific community, since two independent groups demonstrated the same demethylation of H4R3me2, and validated these observations by mass spectrometry. In parallel, our group identified the first non-histone target of JMJD6, namely ERα [143] (Figure 3G). We demonstrated that JMJD6 (i) interacted with methylated ERα (metERα), (ii) was part of the previously described metERα/Src/PI3K complex, and (iii) demethylates metERα. Recently, Lawrence et al. showed that JMJD6 demethylates the RNA helicase A (RHA) in foot-and-mouth disease virus infected cells, in order to facilitate viral replication [144] (Figure 3I). More recently, Gao et al. showed that JMJD6 demethylates the HSP70 (heat-shock protein of 70 kDa) on R469 residue *in vitro* and in cell lines (Figure 3C) [145]. Indeed, JMJD6 knock-down in cells increased the level of methylated HSP70, while the *in vitro* demethylation assay using recombinant JMJD6 protein was confirmed by mass spectrometry. These authors also showed that the demethylation of HSP70 reverses the recruitment of the preinitiation complex (TFIIH) to and expression of the RARβ2 (retinoic acid receptor) gene, the functions of which are driven by the Carm1-methylation of HSP70 [145]. The transcription factor PAX3 is also a potential substrate for JMJD6 [147]. Indeed, the authors demonstrated that the loading of PAX3 onto mitotic chromosomes requires arginine methylation, which is regulated by the catalytic activity of JMJD6.

While a majority of studies have reported that the catalytic activity of JMJD6 is mainly localized in the nucleus, JMJD6 also demethylates cytoplasmic substrates. For example, JMJD6 interacts with and demethylates methylated ERα in the cytoplasm of breast epithelial cells [143]. More recently, a novel cytoplasmic substrate for JMJD6 was identified, namely TRAF6 (Tumor Necrosis Factor (TNF) Receptor-associated Factor 6 (Figure 3H), which is demethylated in response to toll-like receptor ligands in order to regulate the innate immune system [146]

So far most investigations have focused on the involvement of JMJD6 in asymmetric demethylation, with the identification of histone and non-histone substrates like ERα, RHA, HSP70 and TRAF6. The role of JMJD6 in symmetrical demethylation is less clear, since only one substrate was identified, namely histone H4 symmetrically dimethylated on R3 (H4R3m2s) [15, 138]. Our group found that the depletion of JMJD6 does not affect global symmetric dimethylation levels, but impacts global asymmetric dimethylation levels, supporting the idea that JMJD6 preferentially demethylates asymmetric dimethylation. However, even if JMJD6 possesses a JmjC domain, it is clearly not involved in histone lysine demethylation [148].

Finally, we speculate that the controversy around the demethylase activity of JMJD6 came from its low efficiency in demethylating substrates *in vitro*. Several findings concur with this hypothesis. (i) Structurally, JMJD6 shares a JmjC domain similar to lysine demethylases [14, 149], but lacks a consensus domain known to target methylated arginine proteins, as Tudor domain for example. Consequently, JMJD6 may require binding partners to target its substrates. This has also been reported for PRMT5 and for the lysine demethylase LSD1, which requires MEP50 and Co-REST to become fully active [103, 150]. Interestingly, Unoki and colleagues recently demonstrated that JMJD6 requires a partner to recognize modified histones and catalyze their hydroxylation, namely UHRF1 (ubiquitin-like with PHD and RING finger domains) [139]. (ii) Post-translational modifications may also regulate its activity. For example, the hydroxylase activity of JMJD6 is required for its own oligomerization [151, 152], an essential event for its demethylase activity. Of note, we personally observed that the quality/purity of the recombinant protein preparation and the use of fresh demethylation buffer containing Fe (II), 2OG and ascorbate are two important steps to optimize the demethylation assay.

Altogether, these findings clearly demonstrate the bifunctional enzymatic activities of JMJD6, its distribution throughout the cells in both the nucleus and the cytoplasm, and its implication in major biological processes (Figure 3). And although some of these processes have only recently been uncovered, the association between JMJD6 dysregulation and cancer has already been reported.

#### JMJD6 and cancer

Indeed, the expression profile of JMJD6 has been investigated by several groups in different types of cancers (Table 4). Using tissue microarrays, Wang and colleagues found that JMJD6 is upregulated in all types of carcinoma including breast, liver, lung, renal, pancreatic, colon, esophageal, rectal and gastric cancers [21]. However its highest level of expression was found in colon adenocarcinoma, and the authors identified that a high level of JMJD6 expression was associated with a shorter survival time [21]. Furthermore, the expression of JMJD6 expression was confirmed to be higher in lung adenocarcinomas than in normal tissue [22]. Likewise, it was also described that JMJD6 was highly expressed in oral squamous cell carcinoma (OSCC) compared to normal tissues [153]. These authors demonstrated that JMJD6 was positively correlated with oral carcinogenesis, was enriched in oral cancer stem cells (CSC), and was identified as a novel regulator of OSCCs.

In addition several reports have shown that an overexpression of JMJD6 is associated with a poor prognosis in different cancers [22–25], in particular, in lung and oral cancer [22, 25]. In breast cancer, a high level of JMJD6 mRNA expression was linked with tumors with poor outcomes [24, 26], which was confirmed at the protein level, after analyzing 133 breast tumors [23]. Overall these findings strongly indicate that JMJD6 is involved in breast tumorigenesis, and, more precisely, it was shown that the overexpression of the catalytically inactive JMJD6 mutant resulted in a loss of its deleterious effects on proliferation, migration and invasion [23]. In addition, a recent study demonstrated that JMJD6 cooperates with c-Myc to enhance tumorigenesis. Indeed, JMJD6 binds to the p19ARF promoter, where it demethylates H4R3me2a leading to the inhibition of c-Myc-induced apoptosis under various stress conditions [26]. Of note, co-expression of high levels of JMJD6 and c-Myc is associated with poor prognosis for patients harboring ERα-positive breast tumors.

However, we cannot distinguish from the latter study, which of its enzymatic activities is involved in breast tumorigenesis, since the mutated amino acids are required for iron binding, a prerequisite event for both arginine demethylation and lysine hydroxylation. Nevertheless, overexpression of JMJD6 in several cancers may trigger excessive demethylation and/or hydroxylation of several substrates involved in tumorigenesis. For example, JMJD6 demethylates ERα, suggesting that the demethylase activity of JMJD6 could disturb non-genomic estrogen signaling, a pathway activated in aggressive breast tumors [63]. However, JMJD6 has been shown to promote colon carcinogenesis through its hydroxylase activity. Indeed, JMJD6 interacts with the tumor suppressor p53 and catalyzes its hydroxylation. JMJD6 knock-down represses p53-dependent colon cell proliferation and tumorigenesis in mice models [21]. Likewise, JMJD6 depletion results in an alternative splicing of VEGFR, generating a soluble form of the receptor, which inhibits angiogenesis by binding directly to VEGF [138]. These authors demonstrated that JMJD6 mediates alternative splicing of VEGFR by interacting with U2AF65. Since U2AF65 is hydroxylated by JMJD6 [15], we can speculate that the overexpression of JMJD6 may increase angiogenesis *via* its hydroxylase activity, in order to promote tumor proliferation.

In conclusion, the catalytic activities of JMJD6 are involved in cancer tumorigenesis, and we can expect that the discovery of more substrates will emphasize its link with cancer.

**Table 4:** JMJD6 dysregulation in human cancers

Cancer type	Observations	References
**Colon adenocarcinoma**	Protein overexpression is associated with shorter survival time (90 tumors)	[21]
**Lung adenocarcinoma**	Protein overexpression is an independent marker of poor prognosis (154 tumors)	[22]
**Breast cancer**	High mRNA level is associated with poor survival outcome (Supercohort containing 1954 tumors) High levels of expression of JMJD6 are associated with a poor prognosis for patients with tumors expressing high levels of c-Myc (METABRIC database containing 2000 tumors) Overexpression is an independent marker of poor prognosis (133 tumors)	[24] [26] [23]
**Oral squamous cell carcinoma**	Overexpression is an independent marker of poor prognosis (16 tumors)	[153]

### CONCLUSIONS

Protein arginine methylation is a PTM involved in numerous crucial cellular pathways. The altered expression and/or enzymatic activity of the PRMTs and arginine demethylase are involved in breast tumorigenesis. PRMTs are very often overexpressed in tumors, implying that knock-out experiments cannot be used to decipher their molecular impact on cancer development. Furthermore, tools, such as mouse models, are becoming a necessity to determine the precise role of these enzymes in cancer biology. In order to analyze the direct impact of PRMTs on cancer development and progression, mouse models overexpressing a specific PRMT in a defined organ would be essential. Specific inhibitors are also lacking. Indeed, although several PRMT inhibitors have been described, their selectivity and specificity for the different PRMTs are not optimal, and additional experiments need to be conducted to determine the efficacy of these drugs *in vivo* [154]. We can speculate that, similarly to histone deacetylase (HDAC) inhibitors [155], PRMT inhibitors could be used in therapeutic strategies in the future. Because of the clear involvement of arginine methylation in cancer, the development and validation of such drugs in clinical trials may represent the next challenge in this field of research. Additionally, numerous events regulate PRMTs, such as their interaction with some regulatory proteins, miRNAs, or other PRMTs, which could also be targeted to disrupt their enzymatic activities. Likewise, JMJD6 is also overexpressed in several cancers, such as breast, oral, lung and colon cancer [21–25]. Of interest, the COSMIC database referenced a mutation of JMJD6 on H187 residue in kidney carcinoma, which participates in the binding of Fe (II), and could impair its enzymatic activity. However, there are currently no data available to distinguish which of its enzymatic activities is specifically impaired. An effort should be made to understand the regulation of the enzymatic activities of JMJD6 and to develop specific inhibitors of its demethylase and hydroxylase activities. Finally, for arginine methylation, protein interactions with miRNA or PTMs could dysregulate the activity of JMJD6, resulting in tumorigenesis. By improving our understanding of the regulation of JMJD6, we should therefore be able to identify novel ways to target this protein.

Nine PRMTs catalyze ADMA and SDMA modifications, and it is tempting to presume that more arginine demethylases are yet to be identified. This exciting field of research still requires more molecular investigations in addition to physiological analyses to gain a better understanding about how and why arginine methylation is dysregulated in cancer biology.
